# The Potential and Challenges of Proton FLASH in Head and Neck Cancer Reirradiation

**DOI:** 10.3390/cancers16193249

**Published:** 2024-09-24

**Authors:** Chingyun Cheng, Liming Xu, Hao Jing, Balaji Selvaraj, Haibo Lin, Michael Pennock, Arpit M. Chhabra, Shaakir Hasan, Huifang Zhai, Yin Zhang, Ke Nie, Richard L. Bakst, Rafi Kabarriti, J. Isabelle Choi, Nancy Y. Lee, Charles B. Simone, Minglei Kang, Hui Wu

**Affiliations:** 1 Department of Radiation Oncology, Rutgers Cancer Institute of New Jersey, New Brunswick, NJ 08901, USA; chingyun.cheng@rutgers.edu (C.C.);; 2 Department of Human Oncology, University of Wisconsin School of Medicine and Public Health, Madison, WI 53792, USA; 3 Department of Radiation Oncology, Tianjin Medical University Cancer Institute & Hospital, National Clinical Research Center for Cancer, Tianjin’s Clinical Research Center for Cancer, Key Laboratory of Cancer Prevention and Therapy, Tianjin 300060, China; 4 Department of Radiation Oncology, National Cancer Center/National Clinical Research Center for Cancer/Cancer Hospital, Chinese Academy of Medical Sciences and Peking Union Medical College, Beijing 100021, China; 5 New York Proton Center, New York, NY 10035, USA; 6 Department of Radiation Oncology, Albert Einstein College of Medicine and Montefiore Medical Center, Bronx, NY 10461, USA; 7 Department of Radiation Oncology–Radiation Oncology Associates, Icahn School of Medicine at Mount Sinai, New York, NY 10029, USA; 8 Department of Radiation Oncology, Memorial Sloan Kettering Cancer Center, New York, NY 10065, USA; 9 Department of Radiation Oncology, The Affiliated Cancer Hospital of Zhengzhou University, Henan Cancer Hospital, Zhengzhou 450008, China

**Keywords:** proton therapy, FLASH radiotherapy, head and neck cancers, stereotactic body radiation therapy, reirradiation

## Abstract

**Simple Summary:**

Ultrahigh-dose-rate therapy, known as FLASH radiotherapy (RT), is an emerging cancer treatment technique that offers similar tumor control to conventional RT but with the enhanced protection of normal tissue through the FLASH-sparing effect. Preclinical studies on animals and cell lines show promising results. This is significant for patients with recurrent tumors and reirradiation cases, where conventional RT has high toxicity rates. FLASH-RT can potentially improve tumor control while reducing side effects and preserving quality of life. Among the FLASH modalities, proton therapy stands out for its superior dosimetric and delivery characteristics, making it a safe and effective option for human malignancies. Despite its potential, proton Bragg peak FLASH for HN cancer remains underexplored, and this review highlights the novel proton conformal FLASH techniques, which allow for high-quality plans while minimizing radiation exposure to critical organs at risk (OARs) for HN cancer reirradiation.

**Abstract:**

Ultrahigh-dose-rate therapy, also known as FLASH radiotherapy (RT), is an emerging technique that is garnering significant interest in cancer treatment due to its potential to revolutionize therapy. This method can achieve comparable tumor control to conventional-dose-rate RT while offering the enhanced protection of normal tissue through the FLASH-sparing effect. This innovative technique has demonstrated promising results in preclinical studies involving animals and cell lines. Particularly noteworthy is its potential application in treating head and neck (HN) cancers, especially in patients with challenging recurrent tumors and reirradiation cases, where the toxicity rates with conventional radiotherapy are high. Such applications aim to enhance tumor control while minimizing side effects and preserving patients’ quality of life. In comparison to electron or photon FLASH modalities, proton therapy has demonstrated superior dosimetric and delivery characteristics and is a safe and effective FLASH treatment for human malignancies. Compared to the transmission proton FLASH, single-energy Bragg peak FLASH is a novel delivery method that allows highly conformal doses to targets and minimal radiation doses to crucial OARs. Proton Bragg peak FLASH for HN cancer has still not been well studied. This review highlights the significance of proton FLASH in enhancing cancer therapy by examining the advantages and challenges of using it for HN cancer reirradiation.

## 1. Introduction

### 1.1. Challenges in Head and Neck Cancer Recurrence or Reirradiation

Head and neck (HN) squamous cell carcinoma (HNSCC) is a complex and intricate disease involving a wide range of cancers that impact vital anatomical areas such as the larynx, oral cavity, pharynx, hypopharynx, nasal cavity, and salivary glands. Being the seventh most prevalent cancer globally, it presents a serious threat to global health. An estimated 890,000 new cases of HNSCC are identified each year, with about 450,000 of these instances ending in death. These concerning figures highlight the enormous toll that HNSCC takes on patients and healthcare systems, accounting for about 4.5% of cancer diagnoses and deaths worldwide [1,2,3].

Up to 60% of patients with HNSCC present with advanced-stage tumors at diagnosis, making the high incidence of locally advanced disease one of the illness’s most noteworthy features [1,4,5,6]. This creates significant hurdles for prognosis and treatment planning because the survival rates differ greatly based on a number of factors, such as the patient’s general health, the histology, the site of origin and stage of the disease, and the efficacy of the chosen treatment methods. Single-institution research showed that the five-year overall survival (OS) rate for all instances of HN cancers is 60.6%, including 49.0% for oral cavity cancers, 54.8% for oropharynx cancers, 50.0% for hypopharynx cancer, and 63.4% for larynx cancers [7].

Major treatment options for HNSCC include surgery followed by adjuvant radiation therapy or definitive concurrent chemoradiation (CRT), reserving surgery as a salvage therapy [8]. Despite significant advancements in surgical techniques, radiation therapy delivery, and systemic therapy agents, challenges persist in optimizing treatment outcomes while minimizing toxicity. A critical concern in the treatment of HNSCC is the proximity of critical organs at risk (OARs) to the targeted treatment volume. Undesired radiation doses deposited in adjacent OARs can lead to increased toxicity and limit the ability to escalate the radiation dose to the tumor, thereby compromising the treatment efficacy.

Of particular clinical relevance are cases of recurrent HNSCC requiring reirradiation, which pose unique therapeutic challenges due to the high doses of radiation previously delivered. Reirradiation with conventional photon-based 3D conformal RT or intensity-modulated radiation therapy (IMRT) techniques may exacerbate concerns related to cumulative dose overlap and the exit dose, potentially increasing the risk of toxicity. In contrast, emerging modalities such as pencil beam scanning (PBS) proton therapy offer promising advantages in terms of minimizing the dose to OARs while maximizing the dose to the tumor [9,10,11], which is particularly important in the reirradiation setting [12], thereby optimizing treatment outcomes. Intensity-modulated proton therapy (IMPT) delivers highly conformal doses to the target without any exit dose beyond the target.

FLASH radiotherapy (FLASH-RT) is an emerging technique characterized by ultrahigh dose rates (>40 Gy/s) and has demonstrated superior normal tissue sparing and comparable local control compared to conventional-dose-rate radiation therapy regimens in preclinical studies [13,14,15,16,17,18,19,20,21]. Animal models have shown that proton FLASH-RT, in particular, exhibits selective tissue-sparing effects at the beam entrance and Bragg peak regions, offering exciting prospects in terms of minimizing toxicity and improving the therapeutic ratios [13,14,15,16,17,18]. This novel FLASH-RT is appealing in revolutionizing the treatment of challenging cancers in the abdomen [22] and thoracic region, which are typically associated with high toxicity and poor prognoses. Additionally, it offers significant potential to improve outcomes in patients undergoing reirradiation [23]. Favaudon et al. first reported the FLASH effect in human HN cancer in a mice model [13]. Børresen et al. [24] studied electron FLASH-RT in dog patients receiving a single high-dose fraction for oral cancers. Konradsson et al. [25] reported their initial experience in canine cancer patients, including oral targets.

The emergence of FLASH-RT has sparked considerable research efforts to explore its underlying radiobiological mechanisms and optimize its clinical implementation. This includes ongoing investigations into modifying existing treatment systems, optimizing the beam and field properties, developing new hardware and software solutions, and refining biological models to better understand the therapeutic potential of FLASH-RT in the treatment of HNSCC. These efforts hold immense promise in advancing the field of radiation oncology and improving outcomes for HN patients, especially those who need reirradiation.

### 1.2. Potential of Proton FLASH-RT for HN Cancer Treatment

Numerous preclinical studies have shown that delivering radiation therapy at an ultrahigh dose rate of 40 Gy per second significantly reduces the normal tissue toxicity while maintaining equivalent, or sometimes even improved, tumor control [13,14,15,16,17,18,19,20,21]. Small animal studies using electron beams demonstrate promising results in sparing normal tissue, such as the lungs [13,14,15], skin [16,17], brain [18,19], and abdomen [20,21]. The first clinical treatment using an electron beam for a human patient with resistant CD30+ T-cell cutaneous lymphoma showed promising results, with positive outcomes observed in both the tumor and the skin [14]. Despite its potential, the translation of FLASH-RT into clinical practice faces challenges, particularly in achieving precise dose distributions and treating deep-seated tumors with electron beam therapy.

**Figure 1:**
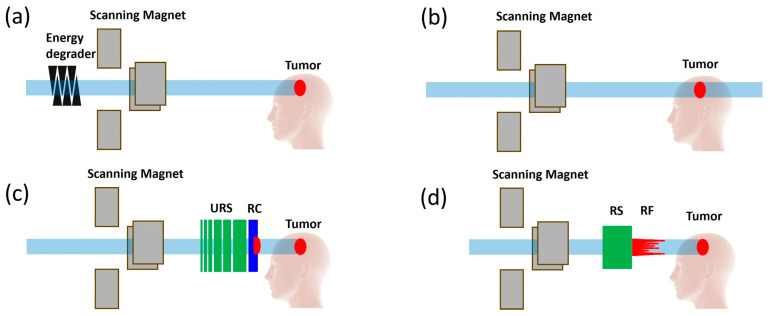
Comparison of pencil beam scanning delivery methods using a cyclotron-based proton system: (**a**) the conventional delivery method utilizing an energy degradation system; (**b**) the transmission or “shoot-through” technique, where the cyclotron operates at its highest energy and beam current to deliver ultrahigh-dose-rate proton therapy; (**c**) the single-energy Bragg peak FLASH technique, which employs a universal range shifter (URS) and range compensator (RC); and (**d**) the conformal FLASH technique, which uses a range shifter (RS) and ridge filter (RF).

**Figure 2:**
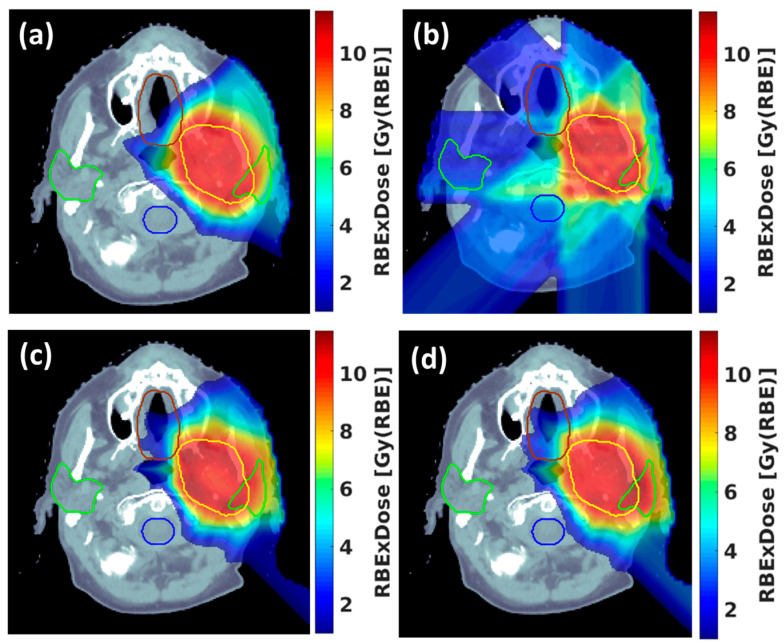
Proton pencil beam scanning (PBS) treatment techniques for conventional-dose-rate and FLASH therapy: (**a**) conventional intensity-modulated proton therapy (IMPT), (**b**) proton transmission FLASH technique, (**c**) single-energy Bragg peak FLASH technique, (**d**) mini-ridge filter technique.

**Figure 3:**
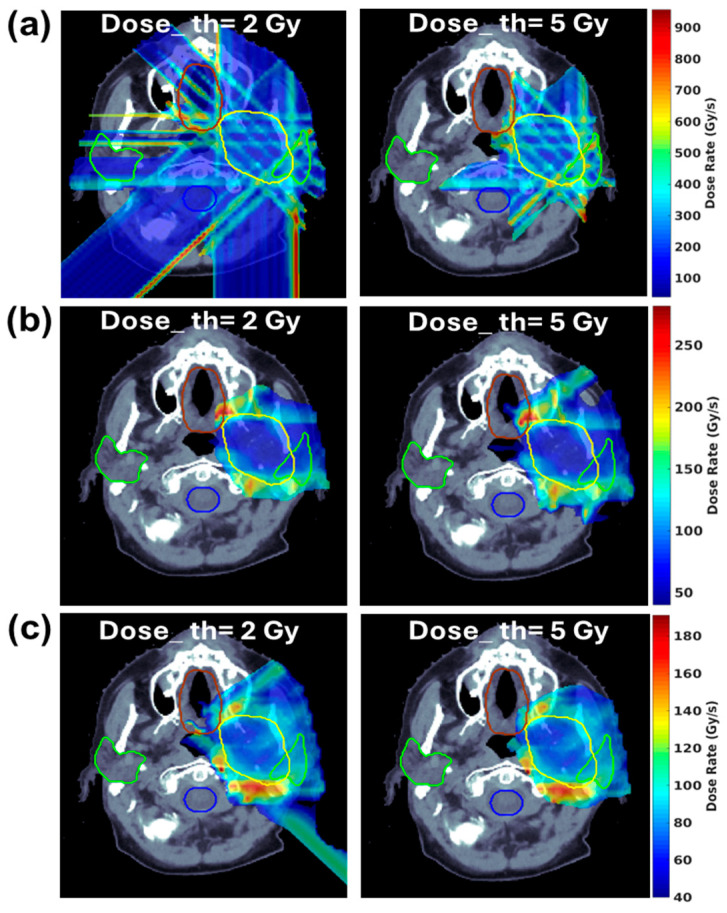
Two-dimensional dose rate distribution for three different techniques for the same representative HN patient in Figure 2, with 2 and 5 Gy dose thresholds applied: (**a**) proton transmission FLASH, (**b**) single-energy Bragg peak FLASH, and (**c**) the mini-ridge filter FLASH method. Here, “Dose_th” represents the dose threshold.

Proton therapy offers superior dose conformality compared to electron beams, making it suitable for the treatment of various tumors [26,27,28,29,30,31]. FLASH-RT usually requires the whole fraction dose delivered within a few hundred milliseconds; the current PBS treatment technique using multiple-energy layers to cover the target from deep to shallow depths is not feasible, as the energy switch time is usually larger than the FLASH delivery time scale. Another limitation in using an energy degrader is that the proton flux/beam current is reduced significantly, which prevents it from reaching the FLASH dose rate. Thus, research interest has emerged in the use of transmission proton beams to shoot through the patient’s body to reach the tumor target [28,29]. Figure 1 illustrates the cyclotron-based proton therapy delivery methods for both conventional-dose-rate RT and FLASH-RT, utilizing transmission, single-energy pristine Bragg peak, and ridge filter (RF) techniques. As shown in Figure 1a, conventional RT employs an energy degrader to adjust the proton beam ranges, delivering the dose layer by layer. By bypassing the degrader and delivering the proton beam at the cyclotron’s highest energy, the beam current can be maintained to achieve ultrahigh dose rates, as depicted in Figure 1b. Since transmission still results in a significant exit dose, researchers have developed single-energy pristine Bragg peak (Figure 1c) and ridge filter (RF) Bragg peak techniques (Figure 1d), incorporating beam-specific pullback devices such as the range compensator (RC) and RF. The conventional IMPT and proton transmission technique using pencil beam scanning are displayed for a representative HN patient in Figure 2a,b. One drawback of the transmission method in proton therapy for FLASH-RT is the exit dose delivered to critical OARs located beyond the target along the beam path, which results in these areas receiving undesirable doses or irradiation. A novel proton therapy technique was developed at the New York Proton Center (NYPC) to deliver conformal FLASH-RT using Bragg peaks [28,32]. Utilizing the Bragg peak characteristics of proton beams, as opposed to the transmission/shoot-through beam method, enables the achievement of the FLASH dose rate while simultaneously minimizing the exit dose beyond the target volume. Figure 2c shows the Bragg peak FLASH dose distribution, which is as conformal as the conventional-dose-rate IMPT in Figure 2a. This approach leverages the potential of the FLASH effect by utilizing the unique physical properties of proton beams to precisely target the dose at a specific depth, thereby minimizing the exposure to surrounding tissue. To achieve ultrahigh dose rates, the Bragg peak FLASH method utilizes the single highest energy output from the cyclotron for the highest beam current. The treatment planning software incorporates an inverse optimization algorithm and the implementation of universal range shifters and range compensators. With proton beams having the highest single-energy and multiple-field inverse optimization, treatment plans at the desired FLASH dose rate for the FLASH-sparing effect are achieved, demonstrating conventional IMPT-equivalent conformality and plan quality. As demonstrated in Figure 2d, the mini-ridge filter technique can also be used to deliver conformal doses to the target [33,34]. This technique employs pin- or blade-shaped range modulators to modulate the pristine Bragg peak, generating a spread-out Bragg peak (SOBP) that achieves uniform dose coverage with a single field. In current practice, some researchers modify the modulation approach to create a non-uniform dose distribution, forming a sparse spot pattern to increase the dose rate [35,36,37]. This method is similar to the single-energy Bragg peak technique, which requires multi-field optimization to achieve a uniform target dose. Figure 3 showcases the 2D FLASH dose rate distribution for all three techniques with 2 and 5 Gy dose thresholds. The color wash highlights the voxels that receive doses of at least 40 Gy/s. Research comparing the dosimetric potential of Bragg peak FLASH proton therapy to PBS transmission FLASH and traditional-dose-rate IMPT in patients undergoing HN reirradiation has been reported by Pennock et al. [38]. The objectives of their study were to evaluate OAR sparing and employ multiple-field optimization to achieve high-FLASH-dose-rate coverage for critical OARs with realistic machine parameters. The results show the potential of Bragg peak FLASH to improve outcomes in HN reirradiation patients by suggesting that it exhibits superior plan quality while retaining adequate FLASH dose rate coverage for critical OARs.

## 2. Current Challenges in Proton FLASH Application

### 2.1. Insufficient Biological Evidence for FLASH Sparing Modeling

Favaudon et al. were the first to study the FLASH effect in human HN cancer HEp-2 xenografts in mice, demonstrating that a 25 Gy dose of FLASH radiation effectively halted tumor growth for 40 days post-treatment, with no visible skin damage in the irradiated area [13]. However, the biology modeling in FLASH protection mechanisms remains limited for HN cancers. The lack of experimental findings linking the clear commencement of the FLASH effect with the tissue-specific dose and dose rate impedes a thorough understanding of this prospective therapeutic modality. It is difficult to define thresholds for the dose and dose rate accurately until the biological processes underlying the FLASH effect are clarified [39]. While studies conducted in vivo and in vitro have shown decreased radiation toxicity in healthy tissue without impairing tumor control, it remains unknown which biological pathways underlie this phenomenon [40,41]. The precise involvement of many factors, including immune-mediated mechanisms [42,43], oxygen deprivation [44,45], and DNA damage [46,47], has been examined and hypothesized, but they have not yet been fully correlated with the FLASH-sparing effect. Furthermore, there is currently a poor understanding of how FLASH-RT affects the dynamic tumor microenvironment, immunological system, and hematological cells in humans. FLASH therapy has therapeutic potential, but, in order to be optimally deployed in clinical settings, further preclinical research and carefully planned human studies are needed to determine its efficacy, safety, and value compared to traditional radiation therapy.

### 2.2. Absence of Optimal Regimens for FLASH-RT

For HN malignancies, stereotactic body radiation therapy (SBRT) is not commonly used. However, treatment in one or just a few fractions is the most well-characterized and currently preferred means of delivering FLASH therapy, as demonstrated in most small animal studies using a single-fraction high dose [13,14,16,17,18,19,20,21,22,23,24,25,48]. Sørensen et al. also studied the repainting effect in FLASH delivery by splitting the total dose into two deliveries, still maintaining the FLASH-sparing effect but with a protection reduction compared with the single-fraction high-dose delivery [26]. Mascia et al. [49] reported the impact of the multiple beams in proton FLASH delivery and found that interruptions in the delivery time can compromise the FLASH-sparing effect in areas of beam overlap. Reirradiation of the HN region has historically been delivered with traditional fractionation and can lead to high rates of acute and late toxicity given the prior high-dose radiation delivered, which can overshadow the possible benefit of retreatment. In general, IMPT is preferred over IMRT because it delivers radiation with a minimal distal dose to surrounding tissue. This property is beneficial in sparing important structures that are located distally to the target region. 

To further improve the therapeutic ratio, SBRT has emerged as a subject of clinical interest for reirradiation. SBRT delivers a higher dose per fraction and offers an overall shorter course of treatment, typically 30 to 40 Gy in three to five fractions [50]. Such a high biologically equivalent dose to small-volume recurrences can often achieve high rates of local tumor control. Despite the risk of late toxicity with an increasing fraction size, numerous studies have reported minimal toxicity associated with SBRT in the management of recurrent HN cancer treatment, indicating its potential as a promising treatment modality [51,52,53,54,55].

Given the role of SBRT and hypofractionation in HN cancer reirradiation, FLASH-RT has provided a similar treatment course but with the potential for lower toxicity due to its sparing effect in these patients. A recent investigation of electron FLASH-RT with a cohort of 11 dog patients receiving a single high-dose fraction for oral cancers determined that, although FLASH-RT was generally effective, high-grade side effects were still commonly reported [24]. Four of the cases had a grade 3 adverse effect on the mucosa or skin at one month after therapy. This rate is similar to what is seen when oral cancers in dogs are treated with traditional fractionated radiation, where severe, acute mucositis is frequently reported. In a randomized phase III trial [56], a single high-dose fraction of 30 Gy FLASH-RT was administered to cats with T1-T2, N0 carcinomas of the nasal planum and compared to the standard 10 × 4.8 Gy conventional-dose-rate RT. Severe late-stage toxicity—specifically, maxillary bone necrosis—occurred in three out of seven cats treated with FLASH-RT between 9 and 15 months post-treatment. In contrast, no cases of maxillary bone necrosis were observed in the cats treated with conventional RT. Simultaneously, the same research group investigated the sparing effect of FLASH-RT on the skin of mini-pigs using large-field irradiation with a single 31 Gy dose. Although no acute toxicity was observed, severe late skin necrosis developed in a volume-dependent manner at 7 to 9 months post-treatment.

The late-stage toxicity in cat trials was due to the administration of a single high-dose beam to the cartilage in one fraction. Even with a significant FLASH effect, late toxicity was anticipated since this is not a clinically appropriate treatment for humans. Most preclinical FLASH data involve single-field, single-fraction treatments [13,16,17,18,19,20,21,42,43,45,46,47,49,50,51,52,53,54,55,57], highlighting the need for more comprehensive studies. Despite this, many groups report 20–30% normal tissue sparing [13,16,17,18,19,20,21], although some see no difference [58,59]. A 20% reduction in human toxicity would be transformative, but it cannot compensate for poor treatment delivery or unrealistic approaches like the one used on cats. Therefore, the new strategy of using Bragg peak FLASH and ensuring that treatment plans are as conformal as standard clinical proton plans are crucial in making this modality viable in clinical settings. Specifically, developing effective treatment protocols for HN cancer reirradiation is of great interest.

### 2.3. Limited Availability of Proton Facilities

The constraints related to the delivery of FLASH-RT are complex and create difficulties in various facets of radiation therapy technology. While electron beams are frequently used in clinical and preclinical FLASH studies, they are not effective in treating deep-seated and large target volumes, thus limiting their application in delivering FLASH-RT to various disease sites. Second, there are substantial technical obstacles to the production of FLASH X-ray beams in the megavoltage energy range, which severely restricts the application of photon FLASH. While it is reasonably easy to achieve ultrahigh dose rates with electron beams using modern linear accelerators, it is quite difficult to transform these electron beams into megavoltage X-ray beams. Typically, electron beams interact with a high-atomic-number target—often tungsten—within the linear accelerator head to produce X-ray beams. However, because of the significant electron heat deposition in the target material, this conversion process is inefficient. Although synchrotron beams offer a different means to produce ultrahigh-dose-rate particles, their availability is restricted by the small number of synchrotron facilities around the world, in addition to the large physical scale, specialized knowledge, and high costs needed to construct, run, and maintain these facilities. Additionally, the current researchers are exploring different approaches, such as redesigning X-ray tubes [60] and improving the linear accelerator target design [61] to achieve FLASH X-rays. These studies, however, are still in their early phases; thus, further study and advancement are required before any real-world application can be achieved [57].

Furthermore, the use of protons for Bragg peak FLASH-RT requires a high beam current and new designs in proton systems. These specifications highlight the vital need for continued development and research to improve proton beam delivery for Bragg peak FLASH treatment applications. Moreover, the development and validation of proton FLASH therapy techniques are made more difficult by the current absence of commercially available treatment planning systems (TPSs) for dosimetry investigations. Dosimetry studies are essential in accurately calculating and tracking the radiation doses given during therapy in order to ensure efficacy and safety. The lack of dedicated dosimetry tools for FLASH therapy presents formidable challenges for researchers seeking to precisely ascertain treatment parameters and optimize treatment regimens.

In conclusion, the development of creative solutions to these technical issues related to providing FLASH therapy requires cooperation amongst several disciplines. To advance FLASH-RT and realize its full potential to enhance patient outcomes in HN cancer reirradiation, certain challenges must be overcome.

## 3. Strategies for Improved Implementation

### 3.1. Design Robust Clinical Trials for HN Cancers

The FAST-01 trial was the first human clinical trial study that focused on evaluating the feasibility of FLASH proton therapy for symptomatic bone metastasis treatment [62]. The trial was an important first step, proving the clinical viability of this novel therapy approach and providing a foundation for further research. Building on the knowledge gathered from FAST-01, the FAST-02 trial is examining FLASH proton therapy’s therapeutic value in more detail, with a particular focus on bone metastases in the chest [63]. It involves a prospective trial (Cincinnati Children’s Hospital) intended to evaluate the toxicities related to FLASH-RT and its efficacy in relieving pain for patients with symptomatic thoracic bone metastases. Despite these developments, the absence of clinical information—particularly with regard to HN cancers—highlights the urgent requirement for thorough clinical investigations.

To determine the suitability and effectiveness of FLASH-RT in treating complex HN cancers, this kind of targeted research is necessary. The development and execution of detailed clinical trials in this area are pivotal in confirming the potential advantages of this innovative treatment and ensuring its incorporation into standard cancer care protocols [63]. Most biological studies have utilized single-fraction high doses, which are not directly applicable to human HN treatments. A potential solution for clinical use is to adopt multiple fields and hypofractionation, which may help to mitigate the long-term toxicities observed in animal trials. Although some studies have suggested that the FLASH-sparing effect might be diminished when using multiple beams [49] or dose repainting [24], further detailed investigations are needed to identify the optimal parameters to achieve the FLASH effect. Additionally, treatment planning and delivery can be optimized and evaluated to meet tissue-specific dose and dose rate thresholds for each field and each fraction.

### 3.2. Development of Innovative Delivery Solutions

Proton FLASH-RT is not widely available due to its expensive infrastructure and high operating costs. Proton therapy necessitates a large financial investment in facilities and specialized machinery. The technological and operational needs of FLASH-RT are even greater, further contributing to its significant costs and limited availability.

To address these challenges and make FLASH-RT more accessible, researchers and innovators have been exploring novel solutions. One of these endeavors is the development of a single-energy Bragg peak FLASH technique for conformal RT, which will eliminate the energy selection and transportation systems, reducing the footprint of the proton systems. This is made simpler by the single-energy Bragg peak FLASH approach, which uses protons at a single energy level and focuses on maximizing the Bragg peak advantages for ultrahigh-dose-rate delivery. This strategy may lessen the complexity and expense of the FLASH-RT equipment required, increasing its viability for a broader range of clinical applications. By reducing the technological barriers and associated costs, the single-energy Bragg peak technique opens up new possibilities for more affordable FLASH-RT [28,64]. As proton therapy remains an underutilized modality [64], and there are geospatial disparities in access to proton therapy [65], this innovation warrants additional study and has the potential to make FLASH-RT available to a larger number of patients. It gives patients with a variety of cancers new hope, even those for whom traditional radiation therapy has considerable risks of adverse effects. It is a step towards democratizing advanced cancer treatments.

### 3.3. Development of Commercial TPSs

Regarding the development of FLASH-RT, one significant barrier is the lack of FLASH dosimetry research for HN malignancies. The main reason for this gap is that there are not many commonly used TPSs that can accurately capture and simulate the unique features of FLASH-RT. The lack of these TPSs makes it more challenging to carry out thorough dosimetric studies, which are essential in comprehending the possible advantages of FLASH-RT, particularly with regard to protecting critical OARs while effectively treating malignancies.

To address this challenge, there is a growing need for vendors to integrate novel FLASH treatment planning techniques into TPSs. In particular, it is thought that integrating ridge filter and single-energy Bragg peak FLASH—two forms of conformal FLASH techniques—into TPSs represents a significant advancement. These techniques represent innovative approaches to modulating the dose distribution of FLASH-RT, potentially offering more precise and effective treatment options for HN cancers. As shown in Figure 2, both techniques can reach at least 40 Gy/s FLASH coverage for 2 and 5 Gy dose thresholds.

Ridge Filter Technique: This approach involves using a physical modulator to shape the proton beam in the depth direction, creating a more uniform dose distribution across the tumor volume [33,34,35,36,37,66], as shown in Figure 1d. The ridge filter is designed to spread out the single-energy Bragg peak over a specific depth range, allowing for the delivery of a high dose to the tumor while minimizing exposure to surrounding healthy tissue.

Single-Energy Bragg Peak FLASH: As discussed earlier, this technique simplifies the delivery of FLASH-RT by using protons at a single energy level to achieve IMPT-like dosimetry quality through multiple-field delivery [28,67]. This method can provide a simpler and more affordable alternative to performing FLASH-RT by reducing the requirement for intricate beam modulation.

More in-depth and advanced dosimetric investigations would be possible when integrating these techniques into TPSs, offering crucial new information about the dosimetric benefits of FLASH-RT. Such investigations are necessary to assess whether FLASH-RT can effectively treat malignancies while sparing critical OARs. Gaining further insight into these areas will enable the field to improve FLASH-RT procedures, thus potentially improving the overall efficacy, safety, and precision of radiotherapy for HN malignancies [68].

The fact that vendors are being encouraged to use these innovative planning strategies emphasizes the greater need for creativity and cooperation in the radiation oncology community. With additional dosimetric studies being conducted and utilizing upgraded TPS capabilities, FLASH-RT’s promise to revolutionize cancer treatment is becoming more and more apparent. This progress not only promises to improve outcomes for patients with HN cancers but also sets the stage for the expansion of the use of FLASH-RT across a wider range of cancers, ultimately contributing to the advancement of cancer care.

## 4. Conclusions

Delivering high doses of radiation therapy in extremely short bursts potentially minimizes damage to healthy tissue while effectively targeting cancerous cells. As such, FLASH-RT could offer a superior therapeutic ratio for HN cancers compared to traditional radiotherapy techniques.

However, translating the promising biological advantages observed in preclinical studies into actual clinical benefits for patients with HN cancers requires a comprehensive and collaborative effort. Such a transition hinges on multidisciplinary research from the bench to the bedside, involving biologists, physicists, clinicians, and engineers. Together, these experts must tackle the technological challenges of delivering FLASH-RT, ensuring its precision and safety, particularly in the complex anatomical regions involved in HN cancers.

Furthermore, conducting thorough clinical trials to evaluate the effectiveness and safety of FLASH-RT accurately is necessary to determine the true clinical benefits of this modality. It is imperative to include regulatory organizations in order to provide unambiguous approval procedures, and patient advocacy groups are vital in providing education and advocating for the availability of this potentially transformative medication.

The journey of FLASH-RT from a novel concept to a standard-of-care treatment embodies the essence of translational medicine, requiring a concerted effort to harness its full potential for the benefit of HN cancer patients. This collective endeavor promises to advance our treatment capabilities and open new avenues to improve patient experiences and outcomes in oncology.
